# Chemical Composition, Physiological and Morphological Variations in *Salvia* subg. *Perovskia* Populations in Response to Different Salinity Levels

**DOI:** 10.3390/ijms252312566

**Published:** 2024-11-22

**Authors:** Zahra Ghaffari, Mehdi Rahimmalek, Mohammad R. Sabzalian, Ahmad Arzani, Razieh Kiani, Shima Gharibi, Katarzyna Wróblewska, Antoni Szumny

**Affiliations:** 1 Department of Agronomy and Plant Breeding, College of Agriculture, Isfahan University of Technology, Isfahan 84156-83111, Iran; zghaffari90@gmail.com (Z.G.); sabzalian@iut.ac.ir (M.R.S.); a_arzani@iut.ac.ir (A.A.); raziehkiani38@yahoo.com (R.K.); 2 Department of Horticulture, College of Agriculture, Isfahan University of Technology, Isfahan 84156-83111, Iran; 3 Department of Food Chemistry and Biocatalysis, Wrocław University of Environmental and Life Sciences, 50-375 Wroclaw, Poland; 4 Core Research Facilities (CRF), Isfahan University of Medical Sciences, Isfahan 81746-73461, Iran; s.gharibi@mail.mui.ac.ir; 5 Department of Horticulture, Wroclaw University of Environmental and Life Sciences, pl. Grunwaldzki 24a, 50-363 Wrocław, Poland; katarzyna.wroblewska@upwr.edu.pl

**Keywords:** *Perovskia*, salinity, physiological response, ionic balance, oxidative damage

## Abstract

This study evaluated the salinity tolerance of five populations of *Salvia* subg. *Perovskia* (*S. abrotanoides* and *S. yadngii*). The aims of the study were to assess essential oil components, as well as growth and physiological parameters of two *Salvia* species in response to salt stress. Four different levels of salinity (0, 60, 90, and 120 mM NaCl) were applied. The effects of various concentrations of NaCl on essential oil content, composition, growth, water relation, proline, lipid peroxidation (MDA), hydrogen peroxide content, and antioxidant enzyme activity, as well as Na and K contents in leaves and the roots were evaluated. The results revealed that root dry weight loss was higher than that of shoots, indicating root vulnerability due to direct exposure to the salt stress. The lowest and highest oil content was obtained in PATKH (0.6%) at 60 mM and PABAD (0.6%) in 90 mM to 2.16% in PABSM population under 120 mM NaCl. Based on GC-MS analysis, 1,8-cineol (11.64 to 22.02%), camphor (2.67 to 27.14%), bornyl acetate (2.12 to 11.07%), borneol (2.38 to 24.37%), β-caryophyllene (3.24 to 7.58%), α-humulene (2.97 to 7.92%), and δ-3-carene (5.31 to 26.65%) were the most abundant compounds. Based on the principal component analysis (PCA), the most salinity-tolerant populations belonged to *P. abrotanoides* species. These populations are characterized by high root stress tolerance index (STI), root elements, and relative water content (RWC) with elevated levels of salinity stress. Finally, the findings might be useful in unraveling the salinity tolerance mechanisms for integrating stress tolerance with medicinal qualities in future studies.

## 1. Introduction

*Salvia abrotanoides* (Kar.) (formerly known as *Perovskia abrotanoides* Kar.) followed by *Salvia yangii* B.T. Drew (formerly known as *Perovskia atriplicifolia* Benth.) have been considered an important Lamiaceae species with different medicinal properties [1], which is a member of the sixth largest family of flowering plants (Lamiaceae) widely distributed in Iran. Wild *Perovskia* can be found in at least five provinces of Iran [2]. *P. abrotanoides* and *P. atriplicifolia* have been used in landscape design, as well as in medicinal plant gardens [3]. These species have been valued for their diverse phytochemicals for human benefits, viz. leishmanicidal [4], antidiabetic [5,6], and antipyretic properties [7].

The salinization of irrigated lands in arid and semi-arid regions is increasing due to improper irrigation and poor soil drainage that leads to the accumulation of exchangeable sodium and soluble salts in the root zone of the soil [8]. Saline soil is difficult to remediate due to the high mobility of the Na and Cl ions in the soil, dynamic nature, and spatial variation in the salt content, which is generated by interactions among the edaphic, agronomic, geographic, and climatic variables [8]. In addition, this is often a short-term and highly costly solution, while the use of salinity-tolerant cultivars is the most efficient way to combat soil and water salinity [9].

Plant growth inhibition due to salinity stress is caused by a variety of factors, among which osmotic and ionic stresses must be considered of primary importance [9,10,11]. The accumulation of Na and Cl ions in plant tissues causes cytotoxicity, which is the most consistent and dramatic effect of salinity. However, Na concentration in plant tissues is the most perceptible and measurable effect, with the main ion interacting with K absorption, although Cl is more dangerous than Na and causes chlorophyll biosynthesis and chlorotic toxicity [12]. Plants use various physiological strategies to deal with excess sodium and chloride, including osmotic adjustment, exclusion of extra Na and Cl from the photosynthetic leaves, and tissue tolerance to Na and Cl accumulation [9].

Some typical effects of salinity and its consequences could be beneficial for the growth and production of ornamental plants. Moreover, the solutions to ameliorate the negative effects induced by saline irrigation on this group of plants are reviewed in an article by García-Caparrós and Lao [13]. There are many reports regarding the effect of salinity stress on growth and physiological properties of the medicinal and ornamental plants including *Echinacea* species [14], *Euonymus japonica* [15], *Lavandula multifida* [16], *Osteospermum* [17], and *Viburnum tinus* [18]. So, evaluation of different salt stress conditions can provide new insights for introducing new tolerant cultivars as well as providing clues for finding the best condition with the highest favorite metabolites such as polyphenolic and alkaloids like tanchinone. To date, no report has been published that examined the response of *Perovskia* species to salinity stress. This report evaluates the effects of various concentrations of NaCl on growth, electrolyte leakage, water relation, antioxidant enzyme activity, as well as Na and K content in leaves and roots of *Perovskia* as well as changes in essential oil compounds at different salinity levels.

## 2. Results

### 2.1. Plant Growth

**Figure 1:**
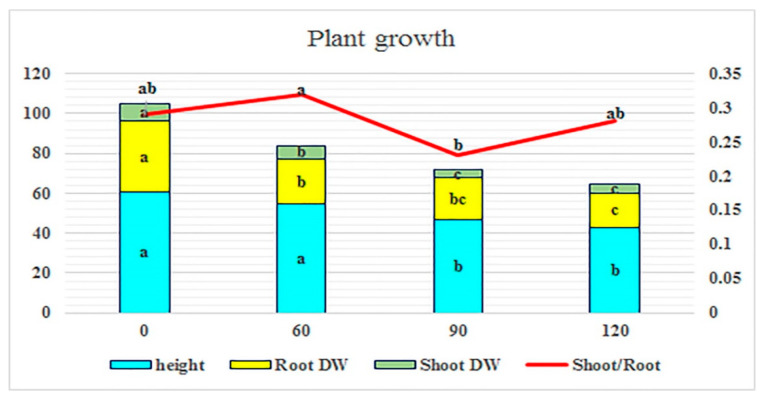
Effect of saline irrigation on plant growth related traits in Perovskia populations. In each column, the means followed by the same letter are not significantly different according to the LSD test at 0.05.

Except for shoot/root dry weight ratio, the effect of salinity stress was statistically significant on all traits studied in relation to plant growth. The genotypes were also significantly different for the dry weight of the shoots and roots. The results of ANOVA also indicated that the genotype × salinity interaction was significant in most of the traits in relation to plant performance (Table 1). The dry weight of both tissues was significantly reduced by elevated levels of salinity. An increasing trend in shoot/root dry weight ratio was observed at the 120 mM level due to the different behavior of genotypes in various saline concentrations. Plants grown at salinity levels of 90 and 120 mM had relatively lower height than those grown under 0 and 60 mM salinity levels (Figure 1).

In terms of root dry weight and shoot/root ratio, no significant differences were shown between populations in 120 mM NaCl. Among *Perovskia* populations, PABSM from Semnan provinces showed the highest mean for root dry weight at 90 mM NaCl. Moreover, in the same salinity conditions, the highest value of the shoot/root ratio was observed in PABAD, PABKH, and PABAY. The highest mean of shoot dry weight belonged to PABKH and PABAY in elevated levels of salinity stress (Table 2).

### 2.2. RWC and EL

**Figure 2:**
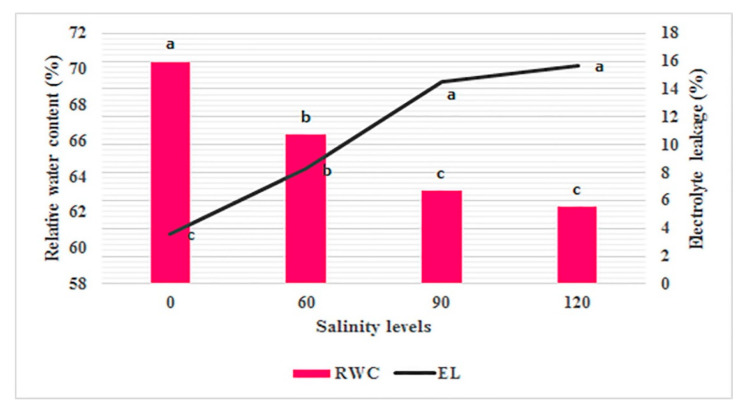
Effect of salinity stress on Perovskia RWC and EL. In each column, the means followed by the same letter are not significantly different according to the LSD test at 0.05.

Regarding RWC and EL (Electrolyte Leakage), the ANOVA results showed significant differences between *Perovskia* populations as well as salinity stresses. Furthermore, a significant genotype × salinity interaction was observed (Table 2). RWC of *Perovskia* plants was reduced by the elevated salinity. Furthermore, salinity stress generally increased EL compared to the normal condition. In terms of RWC and EL, no significant differences were shown between levels of 90 and 120 mM. The highest EL (15.66%) and RWC (70.4%) were recorded in the plants treated with 120 and 0 mM NaCl, respectively (Figure 2). In high levels of NaCl concentrations, PABSM and PABAY showed the highest and lowest means for RWC and EL; and PABKH interestingly acted in the opposite manner (Table 2).

### 2.3. Mineral Concentration (Na, K and K/Na Ratio)

The statistical analysis of the results showed that salinity stress had a significant effect on leaf and root mineral concentration of the *Perovskia* populations excluding root K. The *Perovskia* plants varied significantly in the K content in both tissues, while the Na content of the leaf and root did not vary among the *Perovskia* populations after 76 days of salinity treatment. Furthermore, in most of the traits mentioned, the genotype × salinity interaction was significant (Table 2).

**Figure 3:**
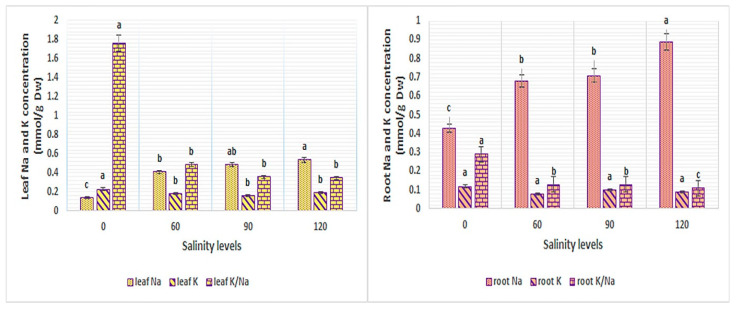
The content of Na and K in relation to NaCl concentration in Perovskia populations. In each column, the means followed by the same letter are not significantly different according to the LSD test at 0.05.

The Na content was positively affected by increasing salinity. On the other hand, the highest leaf Na content was observed in the plants treated with elevated level of salinity in comparison to the control. A similar trend was observed with respect to Na content in the root, except that its value was higher in root than that in leaf (Table 2). The content of K and K/Na ratio in both tissues was gradually reduced with increasing rates of salinity. Indeed, the greatest amount of K and K/Na ratio was recorded in control (Figure 3).

**Figure 4:**
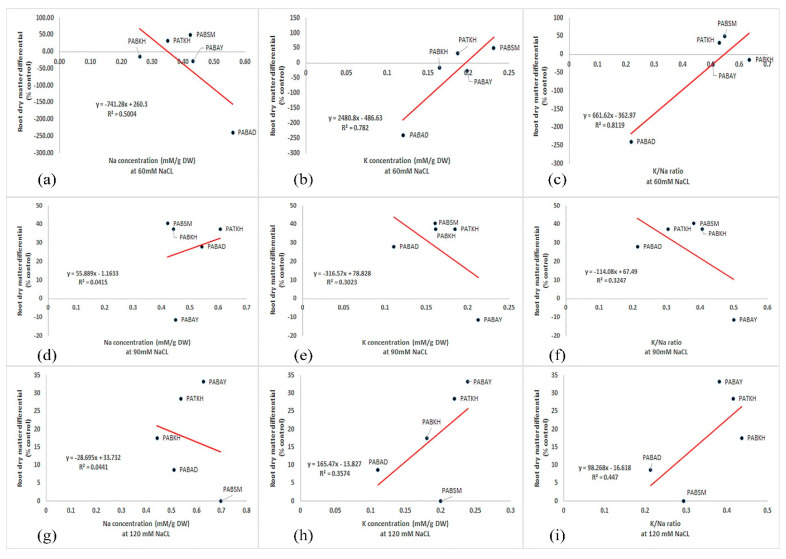
Relationship of root dry weight differential, calculated as a difference between root dry weight in control and saline conditions, with leaf K and Na concentration and the estimated K/Na ratio in Perovskia plants treated with different levels of NaCl: 60 (**a**–**c**), 90 (**d**–**f**) and 120 mM (**g**–**i**).

The relationships between the dry matter produced under various levels of saline conditions and the concentrations of leaf ions were carried out by linear regression analysis. As shown in Figure 4, there was a significant pattern between root dry weight and leaf K, Na concentration, and the estimated K/Na ratio under different salinity levels. In salinity conditions of 60 and 120 mM, the K of the leaf K and K/Na ratio showed positive trends with dry matter in the root, while it was negatively related to Na concentration in the leaf. Moreover, in relation to the 90 mM salinity level, the relationships between root dry matter and leaf ion concentrations were in an opposing manner. At this salinity level, PABSM was the elite genotype for the production of plant biomass and contained the lowest Na concentration in the leaves (Figure 4).

### 2.4. MDA, H_2_O_2_, and Proline Content

**Figure 5:**
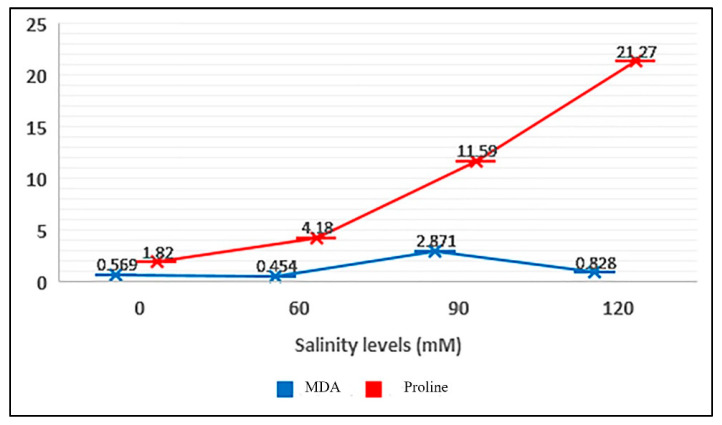
Effects of different salinity levels on proline and MDA contents of Perovskia populations.

The results revealed that the content of malondialdehyde (MDA), H_2_O_2_, and proline was significantly different among the five populations after 76 days of salinity treatment. Furthermore, in these traits, the genotype × salinity interaction was significant (Table 2). The H_2_O_2_ content showed the lowest variation (ranged from 0.35 to 1.45 mM g^−1^ FW), whereas MDA content varied from 0.43 to 2.98 nM g^−1^ FW and proline content ranged from 0.91 to 4.03 μmol g^−1^ FW (Table 3). As shown in Figure 5, the proline and MAD contents increased with the elevated salinity. However, a decreasing trend was observed in the amount of MDA at 120 mM salinity level. In other words, the highest MDA content was recorded in the plants treated with 90 mM NaCl. In the monitored populations, PABAD had the highest proline content and the lowest amount of MDA and H_2_O_2_ in 90 mM NaCl. Conversely, the lowest amount of proline and the highest amount of MDA and H_2_O_2_ were recorded for PABKH (Table 2).

### 2.5. APX and G-POX Activities

**Figure 6:**
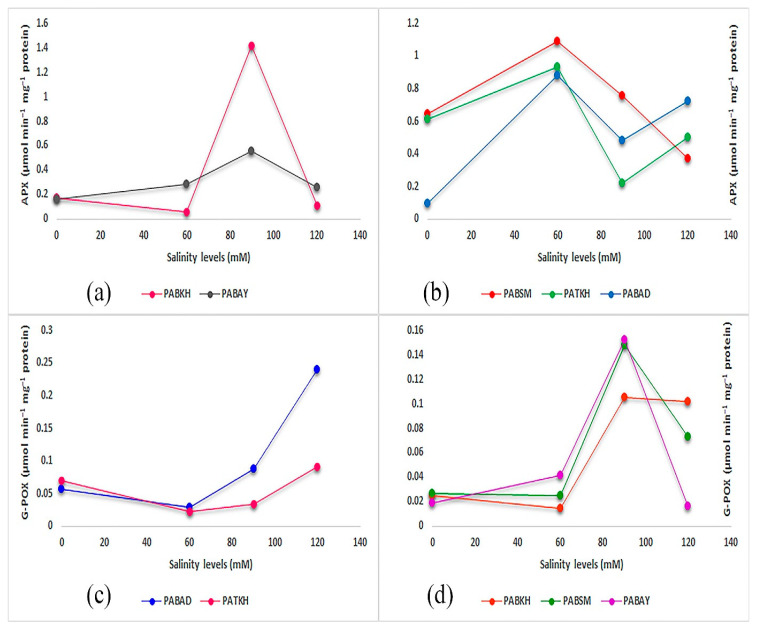
Effect of different levels of salinity treatment on leaf antioxidative enzyme activities in Perovskia populations. (**a**,**b**): L-ascorbate peroxidase (APX) activity; (**c**,**d**): guaiacol peroxidase (G-POX) activity.

The effects of salinity stress on ascorbate peroxidase (APX) and glutathione peroxidase (G-POX) activities are shown in Table 2. The results indicated that the effects of salinity stress, genotype, and their interaction were statistically significant on the activity of antioxidant enzymes. As shown in Figure 6, the highest activity of the measured enzymes was recorded at 90 mM salinity condition for *PABAY* and *PABKH*. Indeed, the trend of enzymatic activity in all investigated salinity stress was similar for the two mentioned populations in both antioxidant enzymes (Figure 6a,d), while the same trend was not obtained in the other populations for both enzymes. Results also revealed that G-POX activity increased as salinity was intensified in *PABAD* and *PATKH*. These populations possessed the same trend for APX activity. Indeed, salinity stress at 60 mM level increased APX activity to its maximum level in *PABSM* and *PABKH* at 90 mM level (Figure 6a,b). Additionally, the highest increase in G-POX activities was recorded in *PABAD* (0.24 µmol min^−1^ mg^−1^protein), *PABSM* (0.14 µmol min^−1^ mg^−1^protein), and PABAY (0.15 µmol min^−1^ mg^−1^protein), respectively (Table 2).

### 2.6. Relationships Between Two Sets of Information (Populations and Traits)

**Figure 7:**
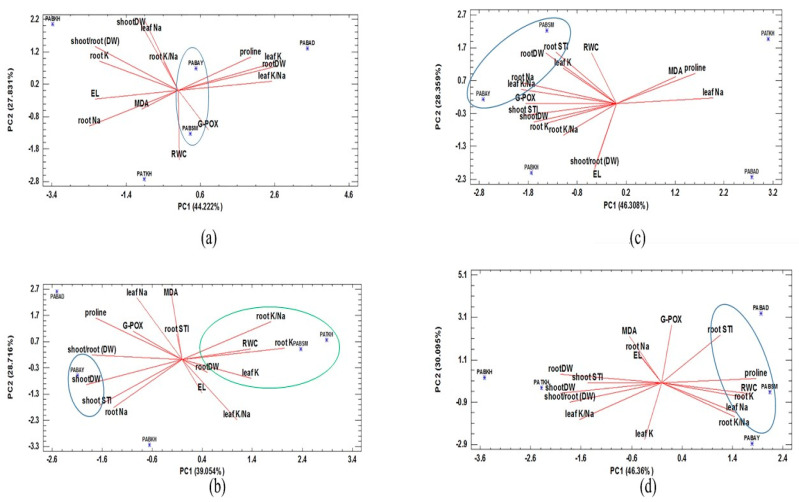
Principal components analysis for classification of five Perovskia population based on the salinity stress tolerance index (STI) and other measured traits at (**a**) 0 mM, (**b**) 60 mM, (**c**) 90 mM, and (**d**) 120 mM NaCl.

Principal component analysis (PCA) was performed to classify the relations between the Salinity Tolerance Index (STI) and most of the traits studied at all examined salinity concentration to distinguish tolerant populations. The results showed that most of the variance in four treatments was approximately more than 70% explained by the first two principal components. Under control conditions, PC1 had positive correlations with leaf K, leaf K/Na ratio, and root dry weight, but a negative correlation with electrolyte leakage. Furthermore, leaf Na and shoot dry weight showed a positive correlation, but RWC displayed negative correlations with PC2. According to this biplot, *PABSM* and *PABAY* were identified as superior populations based on the traits studied (Figure 7a). In 60 mM NaCl condition, *PABAY* based on shoot STI and *PABSM* and *PATKH* based on root elements (such as root K and root K/Na) and dry weight were identified as elite populations (Figure 7b). At elevated levels of salinity, *PABSM* and *PABAY* were also recognized as the most tolerant populations (Figure 7c,d). These populations were characterized by high root STI, root elements, and RWC.

### 2.7. Essential Oil Content

**Figure 8:**
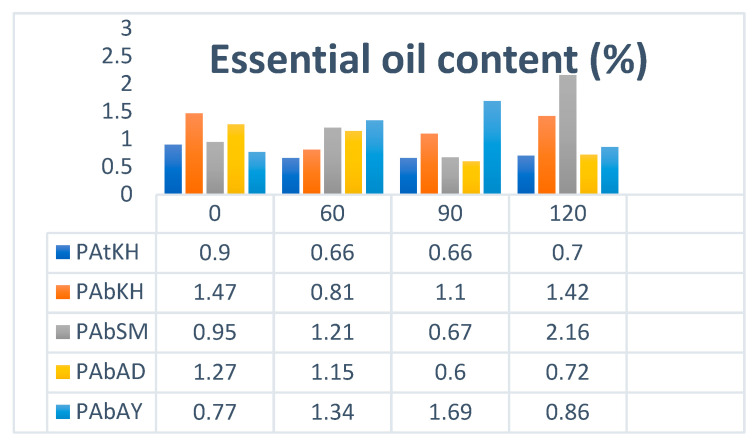
Essential oil content in *Perovskia* population according to salt stress conditions.

*Perovskia* populations showed different responses to salt stress. Accordingly, the lowest and the highest essential oil content was obtained in *PATKH* (0.6%) under 60 mM and PABAD (0.6%) in 90 mM to 2.16% in *PABSM* population under 120 mM NaCl. In general, the 120 mM level of salt stress in most of the populations leads to an increase in the oil content (Figure 8).

### 2.8. EO Chemical Composition

The EO chemical compositions of the studied populations were remarkably dependent on salinity stress and plant species (Table 4). In total, thirty compounds were identified, accounting for 84.03–94.27% of the total composition of essential oils (Table 3). Each concentration of NaCl and control conditions represented different amounts of components (Table 3). The qualitative and quantitative constituents of the essential oil from the aerial parts of the studied population are presented in Table 4. The main compounds were 1,8-cineol (11.64 to 22.02%), camphor (2.67 to 27.14%), bornyl acetate (2.12 to 11.07%), borneol (2.38 to 24.37%), *E*-β-caryophyllene (3.24 to 7.58%), α-humulene (2.97 to 7.92%), and Δ^3^-carene (5.31 to 26.65%) (Table 3). Our results indicated that the EO of the studied populations had different responses to salinity. Additionally, minor compounds had various responses to NaCl concentrations (Table 3). The highest amounts of Δ^3^-carene (20.74%) and borneol (19.15%) were observed at 120 mM NaCl (Table 3). Also, the highest camphor (27.14%) was observed at 90 mM NaCl (Table 3). The essential oils of the studied Perovskia populations were found to be rich in oxygenated monoterpenes (30.17 to 59.94%), while sesquiterpenes ranged only from 10.07 to 21.46.

## 3. Discussion

### 3.1. Effect of Plant Growth

The results revealed that root dry weight loss was higher than that of shoots, indicating root vulnerability due to direct exposure to soil salt. This observation was in line with Sánchez-Blanco et al. [19] report. PABSM and PABAY were tolerant with the least loss of dry matter. In the literature, the reduction of dry matter production and plant height under salt stress in sensitive plants has been considered as a commonly reported occurrence. For example, Tanaka et al. [20] reported a decrease in dry matter production in thyme and oregano after NaCl treatment. They also described the tolerance of basil and sage without a decrease in their dry matter. According to Acosta-Motos et al. [21], reduced fresh or dry weight is mainly due to a decrease in the number of leaves or the formation of smaller leaves and a reduction in plant height. However, this reduction in size could be an advantage for nursery growers because it is both customer-friendly and easier to manage [13]. Previous reports revealed that salinity reduces the absorption of nutrients in plants. In fact, salinity can decrease the accumulation of nitrogen in plants. The decreased absorption of N under saline conditions is due to the interaction between Na and NH_4_ and between Cl^−^ and NO^3−^, which ultimately reduces plant growth and yield [22].

In plants subjected to saline conditions, there are different behaviors with respect to water relation. In fact, due to the intensification of salinity in the rooting medium and the subsequent decline of the water potential in the soil solution, plants must increase their water and osmotic potential to ensure water flow. As a result, it is common to expect the water potential to be negative in plants grown in saline conditions [22,23]. However, an increase in leaf water potential under salinity stress has also been reported by Niu and Rodriguez, [24] in *Teucrium chamaedrys* from Lamiaceae. Our results were in agreement with those that reported the decline in water potential by elevating salinity in Lamiaceae plants [25,26,27]. A decline in RWC indicates a decrease in turgor, which reduces the water required for morphological and physiological processes including cell elongation, stomatal opening, and photosynthesis-related processes [28]. Decreases in leaf RWC may be caused by salt-induced water deficiency, or root systems may not be able to compensate for water transpiration due to reduced adsorption levels [29]. According to Rademacher [30] reports, decreased leaf area due to changes in cell water relationships and turgor pressure can still be beneficial for nursery growers when they need to produce compact plants without consuming growth regulators. Furthermore, to reduce the unfavorable effects of changing the water relations in the plant, an irrigation timetable program based on the demand for evapotranspiration of any species under saline conditions should be used in the absence of salt accumulation in the substrate [13].

Na and Cl are considered as the main ions inducing many physiological disorders and destructive effects in the salinity condition. Sodium also interferes with the absorption of K and ultimately leads to water deficiency induced by disruption of the stomatal adjustment, while Cl is more dangerous than Na because it leads to disorder in chlorophyll production and causes chlorotic toxicity [12]. In PABKH, the level of Na remained low in leaves, while it accumulated in root tissue. This means that the transfer of Na to the leaves is prevented or removed from the roots. In previous studies, this mechanism has been mentioned as a mechanism for salinity tolerance [20,31]. However, in general, the amount of Na in the roots of *Perovskia* was higher than in the leaves and can confirm the observations obtained by Tanaka et al. [20] regarding suppression of Na transfer to the leaves. In PATKH, PABAY, and PABSM, sodium has been accumulated in roots as well as in leaves, unlike in PABKH, where Na in the roots has exceeded the threshold and moved to the leaves. However, especially in PABAY, the K content in the leaves was maintained and decreased in the roots, which suggests the K selectivity in the leaves. In other words, it is a mechanism of response to salinity to retain optimal ion balance against too much entry of Na. As only PABAD showed a large flow of Na from roots to leaves, in addition K has accumulated in root and showed a decline in K content in leaves. PABAD may have suffered from a K shortage during exposure to salinity and has not been able to transmit K to leaves from roots. Our finding was in line with the report by Tanaka et al. [20]. Similarly to the report by Tavakkoli et al. [12] for barley genotypes under salinity stress, different populations of *Perovskia* use various independent mechanisms and different combinations of them for Na and Cl exclusion.

### 3.2. Changes in RWC and EL

Damage caused by abiotic stresses can be seen in the first stage on cell membranes. As a result, the membrane permeability is also affected by the application of salinity stress. Consistent with our findings, salinity stress leads to an increase in EL in summer savory [32]. Indeed, an increase in EL content is a sign of membrane damage and a decrease in its stability, possibly the result of secondary oxidative stress imposed by salinity [33]. Furthermore, monitoring the MDA content can reveal the extent of membrane injury due to salinity-induced oxidative stress [34]. Similar to previous research on Lamiaceae plants such as *Satureja hortensis* L. [32,34], *Ocimum basilicum* [35], *Thymus vulgaris* L., and *Origanum vulgare* L. [20], MDA accumulation was also elevated under salinity stress (Figure 5). Suggesting that *Perovskia* plants have experienced salt-induced oxidative stress, and also by increasing the antioxidant enzyme activity in the plant, the lipid peroxidation of membrane can be prevented [36]. In the literature, a positive correlation has been reported between G-POX activity and MDA level [32]. It is well established that rise in proline content as a compatible solute under saline conditions adjusts the cytoplasmic osmotic potential, protects membranes and enzyme activity as a reservoir of energy and nitrogen [37], and finally it is known as an adaptive mechanism [38]. However, proline accumulation could be used as a selection criterion for salt tolerance due to the existence of their positive relationship [38]. In the study, an increase in proline content is in agreement with other reports on different plants subjected to saline condition [39]. This suggests that exogenous use of proline could also be an effective tool for nursery growers to produce better quality plants applying saline irrigation water [13].

Hydrogen peroxide (H_2_O_2_) production as a reactive oxygen species (ROS) induced by salinity stress can prevent CO_2_ fixation and performs a role of a signaling molecule [13]. The activities of anti-oxidative enzymes also lead to generation and decomposition of H_2_O_2_. Superoxide dismutase (SOD) converts the superoxide radical to H_2_O_2_ and O_2_, while APX and G-POX detoxify H_2_O_2_ into water [20]. The results of the research revealed that due to the low activity of the measured antioxidant enzymes in *PABKH*, the values of MDA and H_2_O_2_ increased and the plant has experienced oxidative damage. In *PABSM* and *PABAD*, the higher activity of antioxidant enzymes efficiently removed H_2_O_2_ and prevented lipid peroxidation, as well as performed a significant role in the salinity stress response. In basil, increased activity of antioxidant enzymes, especially catalase, and subsequently avoidance of lipid peroxidation under salinity stress have been reported [20]. In *PABAY*, the low activity of antioxidant enzymes may be due to low levels of H_2_O_2_ and a lack of membrane damage, because the MDA and EL in this population were at the lowest level. There are also similar reports regarding the response to salinity stress without oxidative damage in rosemary [40]. Although the activity of APX in *PATKH* was high, the concentration of H_2_O_2_ tended to increase. This part of H_2_O_2_ content could have acted as a signaling molecule. Our data were in line with those reported in the literature [20] regarding the anti-oxidative enzyme activity of sage. In the literature, exogenous application of antioxidant compounds has been suggested as a beneficial tool to avoid negative effects of oxidative damage in ornamental plants [13].

### 3.3. Changes on Essential Oil Composition

Most of the previous studies also introduced camphor, 1,8-cineole, borneol, and α-pinene as the main compounds of *Perovskia* essential oils [41,42]. The results are consistent with those obtained by Yu et al. [43] in *Mentha canadensis* L. They reported that salinity stress significantly affects essential oil yield and composition. The present research showed a downward trend in EO content with increasing salt concentration in the soil. However, Karray-Bouraoui et al. [44] observed an enhancement in EO yields in shoots of *M. pulegium* under 50 mM NaCl treatment. They reported that salinity increases both glandular and non-glandular trichome density on both sides of the latter, and young leaves are involved in essential oil production. Farsaraeia et al.’s [45] study on sweet basil showed that salinity stress increased EO content. They reported that the EO content was indirectly influenced by salinity stress through its effects on either the partitioning of the assimilation growth or net assimilation and differentiation processes.

The essential oils of the studied *Perovskia* populations were found to be rich in oxygenated monoterpenes (30.17 to 59.94%), while sesquiterpenes ranged only from 10.07 to 21.46%. These findings are confirmed by those of Sajjadi et al. [46], who reported high amounts of monoterpenes in this genus (78.9%). However, the results of the study by Yu et al. [43] revealed that salinity stress had no effect on oxygenated monoterpenes content, but it increased menthone and pulegone contents, while a decrease in menthol concentration under saline conditions was observed. The higher accumulation of monoterpenes compared to sesquiterpenes may be attributed to the fact that *Perovskia* plants were harvested at the full flowering stage in this study. The elevated monoterpenes in *Perovskia* harvested at the flowering stage might be due to the efficiency of methylerythritol 4-phosphate (MEP) biosynthesis pathway Rahimmalek et al. [10], through which monoterpene synthesis occurs in plastids to provide terpene precursors including isopentenyl diphosphate (IPP) and dimethylallyl diphosphate (DMAPP). These findings are confirmed by those of Stanojevic et al. [47] who reported the variation between the content and composition of the EOs related to the origin of environmental conditions, irrigation plants, species, chemotype geographic distribution, fertilization, season, and harvesting time.

## 4. Materials and Methods

### 4.1. Plant Materials and Growing Conditions

The seeds of five populations of two *Perovskia* species (i.e., *P. abrotanoides* and *P. atriplicifolia*) were planted in February 2016 in 17 × 15 cm pots containing soil with the ratio of 3:1 soil to sand in the experimental field located at Isfahan University of Technology (IUT). These populations were collected from four provinces of Iran and identified using the Flora Iranica by Dr. Rahimmalek. The voucher specimens were deposited in the Herbarium of IUT (Table 4). The research was carried out at Isfahan University of Technology’s research greenhouse, where the average daily temperature was around 25 ± 2 °C and the average night-time temperature was 17 ± 2 °C. The humidity was 60% during experiment and 2000–3000 FC light. The soil was silty clay loam with pH = 7.38 and EC = 3.25 ds/m. To assess salt tolerance of the populations, a randomized complete block design was used to accommodate the two-way factorial experiment, with genotype and salinity as the main factors with three replications. On average, there were three plants per replication and three replicates per population used in each treatment.

### 4.2. Salt Treatments

Two-year-old plants were treated on 21 April 2018 with four concentrations of salinity including: 0, 60, 90, and 120 mM NaCl. The plants were irrigated with salt solution every eight days (1.5 L in each pot). Salinity treatment started with 20 mM NaCl to prevent osmotic shock and progressively increased to reach the desired salinity level in the initial treatment stage. In order to explore the toxicity (second phase of salinity stress) caused by Na accumulation in plant tissues and assure its adequacy, a prolonged duration of salinity treatment, 76 days, was employed.

### 4.3. Essential Oil Extraction

The dried samples (50 g) of shade dried plants (at 25 °C for 4 days) were powdered and mixed with 500 mL of distilled water. Essential oils were isolated using the hydrodistillation method in the Clevenger-type apparatus for 5 h. All chemical compounds were purchased from Phytolab, Vestenbergsgreuth, Germany. 

### 4.4. GC-MS Analysis

An Agilent 7890 gas chromatographer fitted with a fused silica HP-5MS 5% phenylmethylsiloane capillary column (30 m 9 0.25 mm, film thickness of 0.25 lm), coupled to an Agilent mass selective detector 5975 (ionization voltage, 70 eV; electron multiplier energy, 1350 V; and scan range 39–400 *m*/*z*) was applied for analysis. The column temperature was initiated at 60 °C for 4 min, increasing to 260 °C at 4 °C/min; injector and detector temperatures were 290 °C and 300 °C, respectively; carrier gas (helium) flow rate was 2 mL/min; and a transfer line temperature was 200 °C. The compounds were identified by comparing their retention indices (RI) relative to *n*-alkanes (C_5_–C_24_) with those reported in the literature and comparing their mass spectra with those recorded in the mass spectra library of compounds using NIST23 (National Institute of Standards and Technology, Gaithersburg, MD, USA) and Willey (Chem Station data system, Santa Clara, CA, USA). The Adams (2001) library was also used to determine the components.

### 4.5. Plant Growth Parameters

Fresh weight (FW) of the root and shoot tissues was recorded immediately after harvest. Subsequently, the dry weights (DW) of all samples were determined after drying naturally under suitable dry and dark conditions for 72 h. The height was also recorded in the flowering stage. The STI index was calculated according to Fernandez [48] using the following formula:

$$STI=\frac{Yp\times Ys}{Ymp}$$

where Ys and Yp are the dry weights of the sample (root and shoot) under stress and normal conditions, respectively, and Ymp is the mean DW of all genotypes under normal conditions.

### 4.6. Relative Water Content (RWC)

RWC was determined using the method of [49]. The weight of the selected fully expanded and mature leaves (four leaves per replicate) was determined as the total fresh weight (TFW). Subsequently, the turgid weight (TW) was recorded after the leaves were placed in distilled water for 24 h in darkness and environment condition. Before measuring the dry weight (DW), each sample was placed in a paper bag and dried in a 70 °C oven for 48 h. RWC was calculated based on the following formula:

$$RWC(\%)=\left(\frac{TFW-DW}{TW-DW}\right)\times 100$$

### 4.7. Electrolyte Leakage

The fully expanded leaves selected randomly were used to determine electrolyte leakage (EL). The collected leaves were washed with deionized water before being placed in a glass containing 10 mL of deionized water and incubated in darkness and environmental conditions for 24 h. Subsequently, the initial electrical conductivity (EC1) of the solution was determined using an electrical conductivity meter (HI98130 conductivity tester, HANNA, Woonsocket, RI, USA). Subsequently, the leaves were incubated in water bath at 100 °C for 30 min to cause full leakage of ions into the solutions. EC2 was recorded after the solution was cooled down to 25 °C. EL was measured as described by Hniličková et al. (2019) using the following formula:

$$EL\left(\%\right)=(\frac{EC1}{EC2})\times 100$$

### 4.8. Na and Root Na, K Concentrations

After harvesting, the mineral elements (Na and K) were measured in leaf and root in each treatment. An amount of 0.2 g of dry tissues were powdered and used for the preparation of the test solution. Na and K concentrations of the samples were measured after being incinerated in a muffle furnace at 55 °C for 4 h. Before determinations, all ashes were digested with 10 mL of HCl (2 N) solution and the volume of each sample standardized to 100 mL. Na and K concentrations were determined by flame photometry (Jenway PFP7, UK), using a standard curve [50].

### 4.9. Lipid Peroxidation (MDA)

The estimation of malondialdehyde (MDA), a marker of lipid peroxidation, was determined by the thiobarbituric acid (TBA) reaction. Briefly, 300 mg of fresh leaves were quickly frozen and ground with liquid nitrogen, and then homogenized in 5 mL of 0.1% trichloroacetic acid (TCA) and centrifuged at 14,000 rpm for 10 min. Subsequently, 500 µL of the supernatant were mixed with 2 mL of 0.5% TBA in 20% TCA, and the mixture was incubated at 95 °C for 30 min and then immediately chilled on ice. After centrifugation for 5 min at 10,000 rpm, the absorbance of the supernatant was read at 532 and values were subtracted for unspecific absorption at 600 nm. The MDA content was calculated by the extinction coefficient of 155 mM^−1^ cm^−1^ using the formula [51]:

MDA (nM) = ΔA_(532–600)_/1.56 × 10^5^

### 4.10. Hydrogen Peroxide (H_2_O_2_)

For determination of hydrogen peroxide (H_2_O_2_), leaf tissues (300 mg) were ground in liquid nitrogen before being homogenized in 5 mL of trichloroacetic acid (TCA) and then centrifuged at 14,000 rpm for 10 min. Subsequently, 0.5 mL of K-phosphate buffer and 2 mL of reagent (KI, 1 M) were added to 0.5 mL of supernatant. The samples were incubated in darkness for 1 h and the absorbance was measured at 390 nm. The amount of hydrogen peroxide was calculated using a standard curve using the method described by [51].

### 4.11. Measurement of Proline Content

The proline content in leaves was evaluated following the method described by Bates [52]. Approximately 0.5 g of fresh leaves were ground in 10 mL of aqueous 5 sulfosalicylic acid (3%), then centrifuged at 13,000 rpm, 4 °C for 10 min. Subsequently, 2 mL of supernatant was added to 2 mL of acetic acid and 2 mL of acid agent. The mixture was boiled for 1 h in a water bath and then quickly cooled on ice; subsequently, 4 mL of toluene was added to the samples, and then the absorbance of the red layer was recorded at 520 nm. The free proline content was calculated using a standard curve and was displayed as μmol g^−1^ FW.

### 4.12. Measurement of Antioxidant Enzymes

Two different enzyme activities were assessed: (a) guaiacol peroxidase (G-POX) and (b) L-ascorbate peroxidase (APX). The enzyme extract was prepared by grinding 100 mg of fresh leaves with 1 mL of extraction buffer containing sodium phosphate (0.5 M, pH 7.5), polyvinylpolypyrrolidone (PVP, 1%), and 200 μL ethylenediaminetetraacetic acid (EDTA 0.5 M). The homogenates were subsequently centrifuged at 4 °C, 13,000 rpm for 30 min, and then the collected supernatant was used for protein and enzyme assays.

To determine the activity of G-POX, we used the protocol described by Herzog and Fahimi [53]. Their action solution was composed of 100 μL enzyme extract, 100 μL guaiacol, and 100 μL H_2_O_2_ (1%), and the oxidation rate of guaiacol by G-POX was measured at a wavelength of 470 nm for 2 min.

The APX activity was evaluated using the protocol of Nakano and Asada [54]. The oxidation rate of ascorbate by APX for 1 min was measured at 290 nm. The reaction solution was composed of 100 μL enzyme extract, 100 μL ascorbic acid (0.5 mM), 3 mL sodium phosphate buffer (50 mM, pH 7.0), and 100 μL H_2_O_2_ (1%). The blank was a reaction solution with water instead of enzyme extract. The activity of APX and G-POX was expressed in μmol min^−1^ mg^−1^ protein.

The soluble protein content was evaluated as described in Bradford [55] using bovine serum albumin as the standard. A volume of 100 μL of the extracts was added to the 2900 μL of Bradford reagent, and after 30 min in darkness, absorption was recorded at 595 nm.

### 4.13. Statistical Analysis

The data were subjected to the analysis of variance (ANOVA) and mean comparisons using the Least Significant Difference (LSD) method using the SAS software (version 9.4; SAS Institute Inc., Cary, NC, USA). Principal component analysis (PCA) was employed to examine the interrelationships between populations and the traits studied under salinity stress conditions. Grouping analyses were performed on STI index and traits measured using Statgraphics Centurion XVI.II.

## 5. Conclusions

It is important to be aware of effective defense responses in creating tolerance to salinity stress in plants. Overall, in the research, two populations of *P. abrotanoides* species (*PABSM*) were identified as a tolerant genotype at a salinity level of about 12 dS m^−1^, because they possessed higher RWC, proline, leaf K, leaf K/Na, and root dry weight as well as antioxidant enzyme activity. Our results suggested that root biomass, RWC, MDA, and root ions content had the main contributors on response to long term salinity in *Perovskia* plants. These results indicate that further research into the genetics and molecular basis of salinity tolerance using this accession is vital in understanding the underlying molecular mechanisms involved in the salinity tolerance of this species.

## Figures and Tables

**Table 1:** Analysis of variance (ANOVA) for studied traits of *Perovskia* under salinity stress.

Mean squares																		
S.V.	df	x1	x2	x3	x4	x5	x6	x7	x8	x9	x10	x11	x12	x13	x14	x15	x16	x17
Treatment	3	201.21 **	139.65 **	0.105 ^n.s^	12.94 **	766.64 **	0.34 **	0.008 **	0.36 **	3.27 ^n.s^	15.17 **	60.95 **	2.07 **	1.58 **	0.015 ^n.s^	894.42 **	0.31 **	0.02 **
Genotype	4	104.80 **	272.37 **	0.404 **	8.53 **	110.50 **	0.014 ^n.s^	0.004 *	0.019 ^n.s^	31.20 **	4.43 **	12.56 *	1.77 **	2.10 **	0.09 **	83.01 ^n.s^	0.17 **	0.003 **
Gen× treat	12	20.81 ^ns^	167.77 **	0.096 *	7.61 **	138.87 **	0.014 ^n.s^	0.004 **	0.044 **	23.13 **	0.95 ^n.s^	13.05 **	0.45 **	4.35 **	0.03 **	80.17 ^n.s^	0.29 **	0.005 **
Error	40	14.88	11.24	0.037	0.032	3.33	0.011	0.001	0.010	7.88	0.80	3.95	0.15	0.31	0.008	92.50	0.006	0.001
C.V.	-	5.88	31.77	22.70	15.30	18.79	27.15	19.83	15.35	23.89	30.08	25.06	16.64	11.68	18.22	18.82	17.54	12.65

x1: RWC, x2: electrolyte leakage, x3: H_2_O_2_, x4: MDA, x5: proline, x6: leaf Na+, x7: leaf K+, x8: root Na+, x9: root K+, x10: leaf K+/Na+, x11: root K+/Na+, x12: shoot dry weight, x13: root dry weight, x14: shoot dry weight/root dry weight, x15: height, x16: APX, x17: G-POX. df: degree of freedom. ** values are significant at *p* < 0.01. * values are significant at *p* < 0.05. n.s: values are not significant.

**Table 2:** Interaction between genotype and salinity on studied traits of *Perovskia* plants.

Salinity (mM)	Gen.	EL (%)	RWC (%)	MDA nMgFW^−1^	Proline µmolgFW^−1^	H_2_O_2_ mMgFW^−1^	Shoot DW (g)	Root DW (g)	Shoot/Root DW (g)	Shoot Height (cm)	APX μmol min^−1^ mg^−1^ Protein
0	PABAY	0 ^b^	71.70 ^a^	0.20 ^c^	1.67 ^a^	0.88 ^c^	9.72 ^b^	34.68 ^b^	0.28 ^b^	60.33 ^a^	0.16 ^b^
	PABSM	0 ^b^	72.45 ^a^	0.80 ^a^	1.62 ^a^	0.47 ^d^	4.69 ^c^	37.64 ^b^	0.13 ^c^	56.33 ^a^	0.64 ^a^
	PABAD	0 ^b^	67.61 ^a^	0.42 ^b^	9.28 ^a^	0.74 ^d^	9.04 ^b^	58.68 ^a^	0.15 ^c^	58.67 ^a^	0.10 ^c^
	PABKH	9.47 ^a^	67.03 ^a^	0.64 ^a^	3.13 ^a^	1.68 ^a^	14.24 ^a^	23.02 ^c^	0.63 ^a^	58.67 ^a^	0.18 ^b^
	PATKH	8.64 ^a^	73.22 ^a^	0.50 ^ab^	2.15 ^a^	1.31 ^b^	5. 01 ^c^	23.44 ^c^	0.23 ^bc^	66.33 ^a^	0.61 ^a^
60	PABAY	1.45 ^c^	67.49 ^ab^	0.30 ^a^	7.46 ^a^	0.30 ^b^	8.79 ^a^	22.08 ^b^	0.42 ^a^	46 ^a^	0.29 ^c^
	PABSM	12.06 ^a^	66.41 ^ab^	0.46 ^a^	3.75 ^a^	0.26 ^b^	5.65 ^a^	38.25 ^a^	0.15 ^b^	58.33 ^a^	1.09 ^a^
	PABAD	7.507 ^b^	61.27 ^b^	0.72 ^a^	9.25 ^a^	0.73 ^a^	7.33 ^a^	17.27 ^bc^	0.41 ^a^	54 ^a^	0.88 ^b^
	PABKH	15.48 ^a^	61.57 ^b^	0.20 ^a^	2.54 ^a^	0.80 ^a^	7.81 ^a^	22.62 ^b^	0.32 ^ab^	57.67 ^a^	0.06 ^d^
	PATKH	4.74 ^bc^	73.96 ^a^	0.44 ^a^	1.96 ^a^	1.18 ^a^	3.77 ^a^	12.58 ^c^	0.29 ^ab^	57.33 ^a^	0.94 ^a^
90	PABAY	14.77 ^ab^	64.70 ^ab^	0.72 ^c^	4.45 ^c^	0.23 ^c^	8.33 ^a^	27.37 ^a^	0.30 ^a^	50 ^a^	0.56 ^b^
	PABSM	12.52 ^b^	67.20 ^a^	0.66 ^c^	7.42 ^bc^	0.31 ^b^	3.07 ^c^	28.50 ^a^	0.10 ^b^	40.33 ^a^	0.76 ^b^
	PABAD	15.75 ^ab^	62.20 ^ab^	0.62 ^c^	11.05 ^b^	0.75 ^a^	2.89 ^c^	8.75 ^b^	0.33 ^a^	49.33 ^a^	0.48 ^c^
	PABKH	17.42 ^a^	59.28 ^b^	1.69 ^b^	6.72 ^c^	1.88 ^a^	5.56 ^b^	18.70 ^a^	0.31 ^a^	52.33 ^a^	1.41 ^a^
	PATKH	11.99 ^b^	62.61 ^ab^	5 ^a^	26.02 ^a^	0.46^b^	2.26^c^	20.96^a^	0.11^b^	41.67^a^	0.22^d^
120	PABAY	4.13 ^c^	63.17 ^ab^	0.36 ^b^	18.64 ^b^	0.36 ^c^	3.93 ^bc^	14.75 ^a^	0.26 ^a^	38 ^c^	0.26 ^c^
	PABSM	23.19 ^ab^	67.21 ^a^	0.92 ^a^	22.22 ^a^	1.27 ^a^	4.09 ^bc^	16.11 ^a^	0.25 ^a^	36.67 ^c^	0.37 ^c^
	PABAD	16.99 ^b^	62.26 ^ab^	0.98 ^a^	19.31 ^a^	0.85 ^ab^	3.34 ^c^	15.76 ^a^	0.21 ^a^	52 ^a^	0.72 ^a^
	PABKH	28.38 ^a^	58.29 ^b^	0.90 ^a^	5.44 ^d^	0.95 ^a^	6.10 ^a^	20.62 ^a^	0.32 ^a^	42.33 ^bc^	0.11 ^d^
	PATKH	5.60 ^c^	60.57 ^ab^	0.94 ^a^	12.75 ^c^	0.51 ^bc^	5.79 ^ab^	19.16 ^a^	0.36 ^a^	45.67 ^ab^	0.50 ^b^
**Salinity** **(mM)**	**Gen.**	**G-POX** **μmol min^−1^ mg^−1^ protein**		**Leaf** **Na** **mMgDW^−1^**	**Leaf** **K** **mMgDW^−1^**	**Leaf** **K/Na** **mMgDW^−1^**		**Root** **Na** **mMgDW^−1^**	**Root** **K** **mMgDW^−1^**	**Root** **K/Na** **mMgDW^−1^**	
0	PABAY	0.019 ^d^		0.14 ^a^	0.25 ^b^	2.18 ^a^		0.38 ^a^	0.15 ^a^	0.39 ^a^	
	PABSM	0.027 ^c^		0.12 ^a^	0.22 ^bc^	1.77 ^a^		0.41 ^a^	0.12 ^a^	0.39 ^a^	
	PABAD	0.056 ^b^		0.15 ^a^	0.32 ^a^	2.27 ^a^		0.26 ^a^	0.06 ^a^	0.22 ^a^	
	PABKH	0.025 ^c^		0.17 ^a^	0.17 ^c^	1.14 ^a^		0.51 ^a^	0.17 ^a^	0.32 ^a^	
	PATKH	0.070 ^a^		0.13 ^a^	0.19 ^c^	1.47 ^a^		0.56 ^a^	0.09 ^a^	0.15 ^a^	
60	PABAY	0.042 ^a^		0.43 ^ab^	0.19 ^a^	0.51 ^a^		0.76 ^a^	0.08 ^a^	0.10 ^a^	
	PABSM	0.025 ^b^		0.42 ^ab^	0.23 ^a^	0.55 ^a^		0.63 ^a^	0.09 ^a^	0.14 ^a^	
	PABAD	0.030 ^b^		0.56 ^a^	0.11 ^b^	0.22 ^b^		0.65 ^a^	0.07 ^a^	0.13 ^a^	
	PABKH	0.015 ^c^		0.26 ^b^	0.16 ^ab^	0.63 ^a^		0.73 ^a^	0.08 ^a^	0.11 ^a^	
	PATKH	0.023 ^b^		0.35 ^ab^	0.18 ^ab^	0.53 ^a^		0.63 ^a^	0.10 ^a^	0.16 ^a^	
90	PABAY	0.15 ^a^		0.45 ^a^	0.21 ^a^	0.50 ^a^		0.77 ^a^	0.09 ^bc^	0.11 ^b^	
	PABSM	0.14 ^a^		0.42 ^a^	0.16 ^a^	0.38 ^a^		0.82 ^a^	0.10 ^b^	0.12 ^b^	
	PABAD	0.08 ^b^		0.54 ^a^	0.11 ^a^	0.21 ^a^		0.56 ^a^	0.06 ^d^	0.10 ^b^	
	PABKH	0.10 ^b^		0.44 ^a^	0.16 ^a^	0.41 ^a^		0.80 ^a^	0.15 ^a^	0.19 ^a^	
	PATKH	0.03 ^c^		0.61 ^a^	0.18 ^a^	0.30 ^a^		0.65 ^a^	0.07 ^cd^	0.10 ^b^	
120	PABAY	0.02 ^d^		0.63 ^a^	0.23 ^a^	0.38 ^a^		0.74 ^b^	0.12 ^a^	0.16 ^a^	
	PABSM	0.07 ^c^		0.70 ^a^	0.19 ^a^	0.29 ^a^		0.69 ^b^	0.08 ^bc^	0.12 ^b^	
	PABAD	0.24 ^a^		0.51 ^a^	0.11 ^a^	0.21 ^a^		1.07 ^a^	0.10 ^ab^	0.10 ^b^	
	PABKH	0.10 ^b^		0.44 ^a^	0.18 ^a^	0.44 ^a^		0.76 ^b^	0.06 ^c^	0.09 ^bc^	
	PATKH	0.09 ^b^		0.54 ^a^	0.21 ^a^	0.42 ^a^		1.16 ^a^	0.07 ^c^	0.06 ^c^	

Means with letter are statistically significant at 5% level probability.

**Table 3:** Comparison of essential oil compositions of five *Perovskia* populations under different salt stress conditions.

			0					60					90					120				
Compound	RI^exp^	RI^lit^	PAtKH	PAbKH	PAbSM	PAbAD	PAbAY	PAtKH	PAbKH	PAbSM	PAbAD	PAbAY	PAtKH	PAbKH	PAbSM	PAbAD	PAbAY	PAtKH	PAbKH	PAbSM	PAbAD	PAbAY
α Pinene	939	937	3.48	6.94	3.35	4.28	4.66	4.53	7.01	4.73	4.89	5.76	4.62	6.47	3.79	3.51	6.22	4.42	11.76	5.85	3.82	5.81
Camphene	954	951	2.71	4.12	3.36	3.67	2.93	3.10	2.68	4.35	3.42	3.48	3.40	2.93	3.25	2.68	3.46	3.11	3.14	3.84	2.85	3.50
Sabinene	973	974	0.61	1.73	2.81	3.76	0.64	0.65	0.71	1.87	2.01	2.89	0.65	0.80	2.09	0.66	0.54	0.98	0.80	1.83	1.89	1.11
β-Pinene	979	979	1.73	2.01	1.21	1.48	1.47	1.59	1.26	1.62	1.29	1.80	1.90	1.68	1.35	1.08	1.81	1.84	1.52	1.90	1.21	2.14
Δ^3^-Carene	1011	1011	9.09	26.65	5.31	7.21	9.05	8.51	20.02	9.42	9.11	7.11	8.64	18.91	6.65	7.49	9.19	6.35	20.74	8.18	5.50	8.76
*p*-Cymene	1025	1025	0.51	0.80	0	0.67	0	0.49	0.69	0.40	0.95	0.38	0.44	0.51	-	0.46	0	0.64	0.57	0.39	0	0.38
Limonene	1029	1029	4.91	1.55	1.06	1.37	1.02	7.67	1.25	2.02	1.22	1.44	4.25	1.29	0.90	1.22	1.26	5.83	1.55	1.82	0.81	1.32
1,8 Cineole	1031	1032	22.02	19.03	14.88	16.50	12.77	18.44	16.57	14.51	11.93	17.18	17.39	20.87	14.41	11.64	12.97	16.31	15.44	14.32	12.91	17.51
γ-Terpinene	1060	1060	0.32	0.91	0	0.45	0	0.35	0.48	0	0.67	0	0.41	0.52	0	0.32	0.35	0.40	0.51	0	0	0.34
Terpinolene	1087	1088	0.39	0	0	0	0	0.35	0	0	0	0	0.46	0	0	0	0	0	0	0	0	0
Linalool	1097	1099	0.49	1.74	0	0	0	0.89	8.87	0.36	0.34	0	0.35	0.70	0	0	0.48	0.39	0.76	0.36	0	0.44
Camphor	1146	1044	6.08	3.26	25.34	23.85	22.09	6.08	3.09	24.86	21.18	23.15	7.35	2.67	14.27	7.02	27.14	7.69	5.18	15.11	11.39	18.92
Borneol	1171	1070	9.19	2.38	5.99	4.29	3.88	4.17	7.97	4.94	10.62	4.20	7.86	7.91	18.62	24.37	2.41	6.09	6.04	9.13	19.15	6.42
α-Terpineol	1188	1190	0.69	0.85	0.67	0.53	0	0.61	1.66	0	0.43	0	0.60	1.88	0.63	0.67	0	0.58	1.60	0.56	0.56	0
Bornyl acetate	1289	1293	10.39	2.12	3.71	3.43	4.95	7.69	3.57	4.89	4.33	5.22	9.10	3.64	9.68	7.55	4.54	11.07	2.84	7.07	7.23	8.04
α-Terpinyl acetate	1367	1350	2.82	0.80	2.02	2.21	2.75	3.17	1.64	2.43	1.87	2.53	2.84	1.42	2.34	2.01	2.86	3.61	1.04	2.50	2.16	2.72
α-Copaene	1376	1376	0	0	0	0	0	0	0	0.48	0	0.47	0	0	0	0	0	0	0	0	0	0
α-Gurjunene	1408	1409	0.34	0	0.93	0.78	0.87	0.56	0.35	1.05	0.68	1.02	0.87	0	0.87	0.55	1.01	0.56	0	0.91	0.42	0.75
β-Caryophyllene	1419	1419	5.46	3.24	3.92	5.72	6.88	7.45	4.68	4.13	6.40	5.64	6.46	7.44	3.45	5.79	7.34	7.25	7.58	5.08	5.19	4.91
α Humulene	1454	1454	5.13	3.00	3.27	4.72	6.10	6.91	4.60	3.64	5.09	4.75	5.83	7.05	2.97	4.82	5.95	6.84	7.92	4.33	4.50	4.22
Aromadendrene	1463	1440	2.57	0	0	0	0	4.72	0	0	0	0	3.83	0	0	0	0	0	0	0	0	0
α-Guaiene	1482	1439	0	0	0	0	0	0.48	0	0	0	0	0.48	0	0	0	0	0	0	0	0	0
γ-Cadinene	1515	1513	0.59	0	1.42	1.12	1.69	0.55	0.75	1.58	0.88	1.64	1.72	0	1.16	0.49	2.31	0.53	0.25	1.25	0.52	1.19
δ-Cadinene	1526	1524	0	0	2.10	1.41	1.32	0.78	0.52	1.00	1.39	0.83	0.95	0	0.71	1.23	0.94	1.15	0.44	1.12	1.34	0.89
α-Calacorene	1541	1542	0	0	0.49	0	0	0	0	0.37	0	0	0	0	0.53	0.32	0	0	0	0.40	0.29	0
Germacrene B	1562	1557	0	0	0.70	0.50	0	0	0	0.52	0.35	0.51	0	0	0.38	0.52	0	0	0	0.49	0.53	0.44
Humulene epoxide	1605	1604	1.84	0.33	0.96	1.20	1.58	1.67	0.93	0.89	0.99	1.05	1.39	1.04	0.86	1.29	0.93	1.65	0.89	0.89	1.46	1.21
β-Oplopenone	1608	1606	0	0	0	0	0	0	0	0	0	0	0	0	1.01	0	0	0	0	0	0	0
γ-Eudesmol	1631	1631	1.31	2.59	0.54	0.66	1.16	1.33	5.25	0	0	0	0.38	5.12	0.70	0.61	0	0.48	2.38	1.45	1.23	0.39
*epi*-α-Cadinol	1652	1640	1.58	0.64	2.13	2.12	1.43	0	2.57	1.73	1.17	0	0.63	1.51	1.50	1.21	0	0.51	2.13	1.86	0	0
Total of compounds	-	-	94.27	84.03	91.26	90.55	85.24	92.74	90.77	91.57	91.52	92.25	92.80	89.31	91.35	88.62	90.72	90.39	93.25	90.07	86.24	93.99
Monoterpene hydrocarbons	-	-	23.75	44.70	17.09	22.87	19.78	27.24	34.10	24.41	23.54	22.68	24.76	33.10	18.03	17.42	22.83	23.57	40.58	23.81	16.07	23.36
Oxygenated monoterpenes	-	-	51.69	30.17	52.61	50.80	46.43	41.04	43.36	51.98	50.69	52.27	45.49	39.08	59.94	53.25	50.40	45.75	32.91	49.05	53.40	54.05
Sesquiterpenes hydrocarbons	-	-	14.09	6.23	12.81	14.24	16.85	21.46	10.90	12.76	14.78	14.84	20.14	14.49	10.07	13.73	17.55	16.33	16.19	13.57	12.79	12.39
Oxygenated sesquiterpenes	-	-	4.73	3.55	3.62	3.97	4.17	2.99	8.74	2.62	2.15	1.05	2.40	7.66	4.07	3.10	0.92	2.63	5.39	4.20	2.69	1.60

RI^exp^: experimental retention indices on HP-5MS column, RI^lit^: literature retention indices (NIST23).

**Table 4:** Herbarium voucher specimen number and origin of the studied Perovskia populations.

Location	Accession Name	Herbarium Voucher Specimen No.
Abyaneh-Isfahan	PABAY *	13356
Shahrud-Mehmandust-Semnan	PABSM	13359
Abardeh-Khorasan Razavi	PABAD	13364
Khash-Sistan and Baluchestan	PABKH	13366
Khash-Sistan and Baluchestan	PATKH	13367

* the first letter means Perovskia, the next two letters show species name, the last two letters show the code of region.

## Data Availability

Data will be available based on the request.
